# Novel Plasma Proteins in Nepalese School-aged Children are Associated with a Small Head Size at Birth

**DOI:** 10.1038/s41598-018-24640-4

**Published:** 2018-04-23

**Authors:** Sun Eun Lee, Keith P. West, Robert N. Cole, Kerry J. Schulze, Lee S.-F. Wu, James D. Yager, John Groopman, Parul Christian

**Affiliations:** 1 Center for Human Nutrition, Dept. of International Health, Johns Hopkins Bloomberg School of Public Health, Baltimore, MD 21205 USA; 2 Mass Spectrometry and Proteomics Facility, Department of Biological Chemistry, Johns Hopkins School of Medicine, Baltimore, MD 21205 USA; 3 Department of Environmental Health and Engineering, Johns Hopkins Bloomberg School of Public Health, Baltimore, MD 21205 USA

## Abstract

Fetal growth restriction increases the risk of poor childhood growth and development and chronic disease in adulthood. Yet, little is known about biological pathways that mediate the long-lasting effects of suboptimal intrauterine growth. We explored the plasma proteome in a cohort of 500 Nepalese children 6–8 years of age to identify plasma proteins associated with multiple anthropometric size indicators at birth. Among 982 proteins analyzed, no proteins differed by birth weight, length, or weight-for-length indicators. However, 25 proteins were differentially abundant in children with a small vs normal head circumference at birth (<−2 vs. ≥−2 z-scores of the WHO growth standards). Angiopoietin-like 6 was 19.4% more abundant and the other 24 proteins were 7–21% less abundant in children with a small vs normal head circumference at birth, adjusted for potential confounders. The less abundant proteins included actins, actin filament organizing proteins (α-actinin, talin, filamin, cofilin, profilin, and vinculin), proteins involved in muscle contraction, and glycolytic enzymes, which were all positively correlated with each other. A novel cluster of childhood plasma proteins involved in angiogenesis and cytoskeleton dynamics was associated with a small head size at birth. The prognostic value of an altered proteomic phenotype remains to be investigated.

## Introduction

Small size at birth represents a major public health burden in South Asia, where 45% of infants are born small for gestational age and 26% of neonates are low in birth weight^1^. Suboptimal intrauterine growth in this region, often attributed to short maternal stature or malnutrition during pregnancy^2^, may increase postnatal risks of infant mortality^3^, stunted childhood growth^4^, poor cognitive development^5^, and chronic disease later in life^6^. These short- and long-term health consequences suggest that nutritional insults during the highly sensitive and critical period of fetal development may result in systemic and permanent modifications of gene expression, cell size and number, and organ structure and function that can adversely affect health outcomes throughout life^7^. However, our understanding of biological processes that are affected by poor fetal development and maintained in postnatal life remains incomplete.

A comprehensive analysis of tissue or circulating proteins using a comparative proteomics approach may help to reveal pathophysiological pathways or associated biomarkers of phenotypes altered by intrauterine growth retardation (IUGR). For example, experimental animal studies have demonstrated that IUGR changed protein expression in liver, muscle, kidney, and small intestines, contributing to abnormal absorption and metabolism of nutrients in newborn pigs and rats^8,9^. Other studies have shown that prenatal undernutrition affects hypothalamus and brain proteomes that may disturb energy and redox homeostasis and brain plasticity and maturation in newborn or adult rats^10,11^. A limited number of human studies have reported differentially abundant serum proteins in umbilical cord- or venous blood samples between IUGR and non-IUGR neonates^12–14^, revealing that differential protein biomarker abundances can be detected in the circulatory system shortly after birth. However, to our knowledge, no human studies have evaluated the persistence of differential plasma protein expression of IUGR into childhood or assessed such differences by multiple anthropometric size indicators at birth.

We assessed nutritional and health status of a cohort of children born to mothers who had participated in a micronutrient supplementation trial in south eastern Nepal with birth measurements^15,16^. Using a quantitative proteomics approach, we previously revealed in this cohort that suites of plasma proteins were associated with various nutritional and health conditions, including status of multiple micronutrients^17–20^, body size and composition^21^, inflammation^22^, and subsequent cognitive function in a subset of children from this cohort^23^. In this study, we test the hypothesis that 6–8-year-old children who had been born small differ in their plasma protein profiles from those of normal birth size.

## Results

### Birth anthropometry of study participants

Birth measurements of study children (n = 500) are summarized in Table 1. Average (SD) weight, length, head circumference at birth were 2.67 (0.41) kg, 47.6 (2.2) cm, and 32.7 (1.3) cm, respectively. Percentages of children who were born stunted, underweight, and wasted (length-for-age [LAZ], weight-for-age [WAZ], and weight-for-length [WLZ] z-scores < −2) were 16.3%, 26.0%, and 18.1%, respectively, and 20.3% of children were born with small head circumference (head circumference-for-age z-scores [HCZ] < −2). Children were, on average (SD), 7.5 (0.4) years old at the time of blood draw. Characteristics of children and household can be found in the Supplementary Table S1.

**Table 1:** Anthropometric characteristics of children at birth for proteomics analysis (n = 500).

	Value
**Birth measurements**	
Weight, kg	2.67 (0.41)
Length, cm	47.6 (2.2)
Head circumference, cm	32.7 (1.3)
Gestational age, week	38.9 (2.9)
**Small birth size**, **%**	
Stunted (length-for-age z-score^a^ < −2)	16.3
Underweight (weight-for-age z-score < −2)	26.0
Wasted (weight-for-length z-score^a^ < −2)	18.1
Small head (head circumference-for-age z-score^a^ < −2)	20.3

Data are expressed as mean (standard deviation) or %. Anthropometry z-scores were calculated based on the World Health Organization growth standards^50^. ^a^Implausible or unavailable z-scores for length-for-age (n = 1), weight-for-length (n = 42), and head circumference-for-age (n = 3) were excluded^51^.

### Differentially abundant plasma proteins between children born with small versus normal sizes

**Figure 1:**
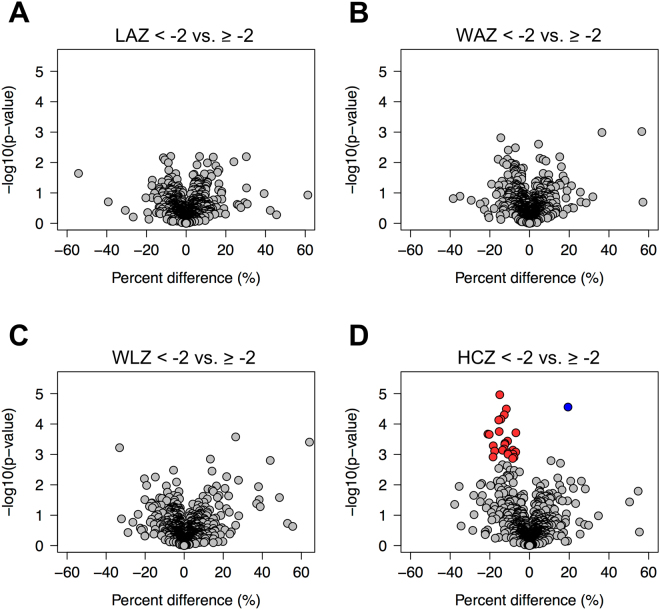
Volcano plots of differentially abundant plasma proteins in children born with small versus normal sizes. (**A**) Length-for-age, (**B**) Weight-for-age, (**C**) Weight-for-length, and (**D**) Head circumference-for-age z-scores <−2 versus ≥−2 (n = 500). All 982 proteins quantified by mass spectrometry in >10% of children were plotted based on corresponding percent differences in relative abundance (x-axis) and −log_10_(p-values) (y-axis) estimated by using linear-mixed effect models adjusted for child age, sex, height, body mass index, ethnicity, caste, schooling, maternal age, parity at the 1^st^ trimester, gestational age, and household wealth index. Proteins passing the pre-determined significance cut-off (q < 0.05) were colored in blue and red for more and less abundant proteins, respectively, in children with small compared to normal birth sizes. Abbreviations: HCZ, head circumference-for-age z-score; LAZ, length-for-age z-score; WAZ, weight-for-age z-score; WLZ, weight-for-length z-score. Implausible or unavailable z-scores of children were excluded in analyses for LAZ (n = 1), WLZ (n = 42), and HCZ (n = 3)^51^.

The relationships between each birth size indicator and all 982 proteins detected in >10% of children (n > 50) are shown in the four panels of volcano plots (Fig. 1). Percent differences (%) in relative abundance of proteins (x-axis) were estimated, adjusted for potential confounding factors including child age, sex, height, body mass index, ethnicity, caste, schooling, maternal age, parity at the 1^st^ trimester, gestational age, and household wealth index. Proteins that are more or less abundant by birth size passing the pre-determined significance cut-off (q < 0.05) are colored in blue and red, respectively. No proteins were differentially abundant based on being stunted (LAZ < −2), underweight (WAZ < −2) or wasted (WLZ < −2) at birth (all q > 0.05) (Fig. 1A–C); however, 25 proteins were differentially abundant in those born with a small versus normal head circumference (HCZ < −2 vs. ≥−2) (Fig. 1D). Among these proteins, angiopoietin-like 6 (ANGPTL6) was 19.4% more abundant (q = 0.0094) and the remaining 24 proteins were 7~21% less abundant (all q < 0.05) in children born with a small versus normal sized head, all adjusted for multiple covariates (Table 2).

**Table 2:** Plasma proteins differentially abundant between children born with small head size and children born with normal head size (head circumference-for-age z-scores < −2 or ≥ −2), q < 0.05.

Protein name^a^	Gene symbol	n for HCZ ≥ −2^b^	n for HCZ < −2^b^	% Difference (95% CI)^c^	p-value^d^	q-value^e^	Accession^f^
**More abundant proteins**							
Angiopoietin-like 6	ANGPTL6	148	47	19.4 (9.8, 29.7)	2.76 × 10^−5^	0.0094	29893555
**Less abundant proteins**							
14-3-3 protein zeta/delta	YWHAZ	294	78	−15.0 (−21.0, −8.6)	1.09 × 10^−5^	0.0094	21735625
Talin 1	TLN1	393	97	−11.7 (−16.7, −6.4)	3.20 × 10^−5^	0.0094	223029410
Alpha-actinin-1	ACTN1	370	92	−12.7 (−18.3, −6.8)	4.99 × 10^−5^	0.0109	194097352
Transgelin-2	TAGLN2	327	87	−14.5 (−20.8, −7.6)	6.96 × 10^−5^	0.0109	4507357
SH3 domain-binding glutamic acid-rich-like protein 3	SH3BGRL3	373	89	−15.5 (−22.3, −8.2)	7.38 × 10^−5^	0.0109	13775198
Tropomyosin alpha-4 chain isoform 2	TPM4	303	82	−15.3 (−22.4, −7.6)	1.77 × 10^−4^	0.0195	4507651
Moesin	MSN	339	88	−6.9 (−10.3, −3.3)	1.94 × 10^−4^	0.0195	4505257
Tropomyosin alpha-3 chain	TPM3	114	39	−21.0 (−30.3, −10.4)	2.14 × 10^−4^	0.0195	114155146
Phosphoglycerate kinase 1	PGK1	103	30	−20.3 (−29.4, −10.0)	2.20 × 10^−4^	0.0195	4505763
Glyceraldehyde-3-phosphate dehydrogenase	GAPDH	390	100	−11.1 (−16.7, −5.1)	3.64 × 10^−4^	0.0293	7669492
Protein S100-A9	S100A9	396	101	−12.4 (−18.7, −5.7)	4.33 × 10^−4^	0.0319	4506773
Cofilin-1	CFL1	266	72	−12.5 (−18.8, −5.7)	4.72 × 10^−4^	0.0321	5031635
Adenylyl cyclase-associated protein 1	CAP1	139	40	−18.3 (−27.2, −8.3)	5.21 × 10^−4^	0.0329	5453595
Actin, alpha skeletal muscle	ACTA1	292	75	−12.8 (−19.4, −5.6)	6.63 × 10^−4^	0.0379	4501881
Gelsolin	GSN	277	76	−8.4 (−13.0, −3.6)	7.22 × 10^−4^	0.0379	38044288
Vasodilator-stimulated phosphoprotein	VASP	264	63	−13.6 (−20.7, −5.9)	7.28 × 10^−4^	0.0379	4507869
Myosin light polypeptide 6	MYL6	243	57	−17.6 (−26.4, −7.7)	7.74 × 10^−4^	0.0380	17986258
Calreticulin	CALR	265	72	−7.0 (−10.8, −2.9)	8.36 × 10^−4^	0.0389	4757900
Profilin 1	PFN1	368	96	−10.9 (−16.7, −4.6)	8.99 × 10^−4^	0.0398	4826898
Filamin-A	FLNA	396	101	−8.4 (−13.0, −3.5)	9.84 × 10^−4^	0.0398	116063573
Peptidylprolyl isomerase A	PPIA	278	75	−10.9 (−16.8, −4.5)	9.88 × 10^−4^	0.0398	10863927
Parvin, beta	PARVB	235	63	−18.4 (−27.9, −7.7)	0.0012	0.0458	20127528
Vinculin	VCL	396	101	−7.8 (−12.3, −3.1)	0.0012	0.0458	4507877
Beta actin	ACTB	396	101	−8.5 (−13.4, −3.4)	0.0014	0.0481	4501885

Abbreviations: HCZ, head circumference z-scores. ^a^Proteins are listed in direction of association and increasing order of q. ^b^Data were missing for HCZ (n = 3). ^c^Percent difference (95% confidence interval) in relative abundance of protein between children born with small compared to normal head circumference adjusted for child age, sex, height, body mass index, ethnicity, caste, schooling, maternal age, parity at the 1^st^ trimester, gestational age, and household wealth index. ^d^*P* value was calculated by testing a null hypothesis of no difference in protein relative abundance between two groups. ^e^Multiple hypothesis testing was corrected using false discovery rate^53^. ^f^GenInfo sequence number as assigned to all nucleotide and protein sequences by the National Center for Biotechnology Information at the National Library of Medicine, NIH.

The results of over-representation analysis showed that 6 annotation terms of the Gene Ontology (GO) database were 3–12-fold significantly enriched in the list of proteins associated with a small sized head at birth over the expected proteins in the reference list (all Bonferroni-corrected *P* < 0.05) (Table 3). The enriched GO terms were *structural constituent of cytoskeleton* (GO:0005200; *P* = 2.0 × 10^−8^) and *actin binding* (GO:0003779; *P* = 4.2 × 10^−3^) in Molecular Function, *actin cytoskeleton* (GO:0015629; *P* = 1.9 × 10^−6^) and *intracellular* (GO:0005622; *P* = 8.1 × 10^−4^) in Cellular Component, and *cellular component morphogenesis* (GO:0032989; *P* = 3.5 × 10^−2^) and *muscle contraction* (GO:0006936; *P* = 5.0 × 10^−2^) in Biological Process ontologies.

**Table 3:** Over-represented Gene Ontology categories in the list of proteins differentially abundant between children with small and normal head circumference (head circumference-for-age z-scores < −2 or ≥ −2) at birth, compared to the reference protein list.

Categories (Gene Ontology ID)	Ontology	Num. in reference list^a^	Num. in analyzed list^b^	Expected num.^c^	Fold enrichment^d^	*P*-value^e^
Structural constituent of cytoskeleton (GO:0005200)	MF	66	14	1.81	7.73	2.00 × 10^−8^
Actin cytoskeleton (GO:0015629)	CC	30	9	0.82	10.93	1.90 × 10^−6^
Intracellular (GO:0005622)	CC	191	15	5.24	2.86	8.13 × 10^−4^
Actin binding (GO:0003779)	MF	25	6	0.69	8.75	4.24 × 10^−3^
Cellular component morphogenesis (GO:0032989)	BP	64	8	1.76	4.55	3.52 × 10^−2^
Muscle contraction (GO:0006936)	BP	12	4	0.33	12.15	4.97 × 10^−2^

Abbreviations: BP, biological process; CC, cellular component; GO, gene ontology; HCZ, head circumference z-scores; MF, molecular function. An analyzed proteins list includes 25 proteins that are differentially abundant between children born with small head size (HCZ < −2) and children born with normal head size (HCZ ≥ −2) at birth (q < 0.05). A reference list includes 909 proteins which were mapped to annotation data (PANTHER GO-Slim) among all proteins detected and quantified by mass spectrometry (n = 982)^54^. Categories are listed in increasing order of p-value. ^a^The number of proteins in the reference list that map to the particular annotation data category. ^b^The number of proteins in the analyzed list that map to the particular annotation data category. ^c^The number of proteins that would be expected in the analyzed list for the particular annotation data category, based on the reference list. ^d^Fold Enrichment of the proteins observed in the analyzed list over the expected number. ^e^*P*-value determined by the binomial statistic after Bonferroni-correction for multiple testing^56^.

**Figure 2:**
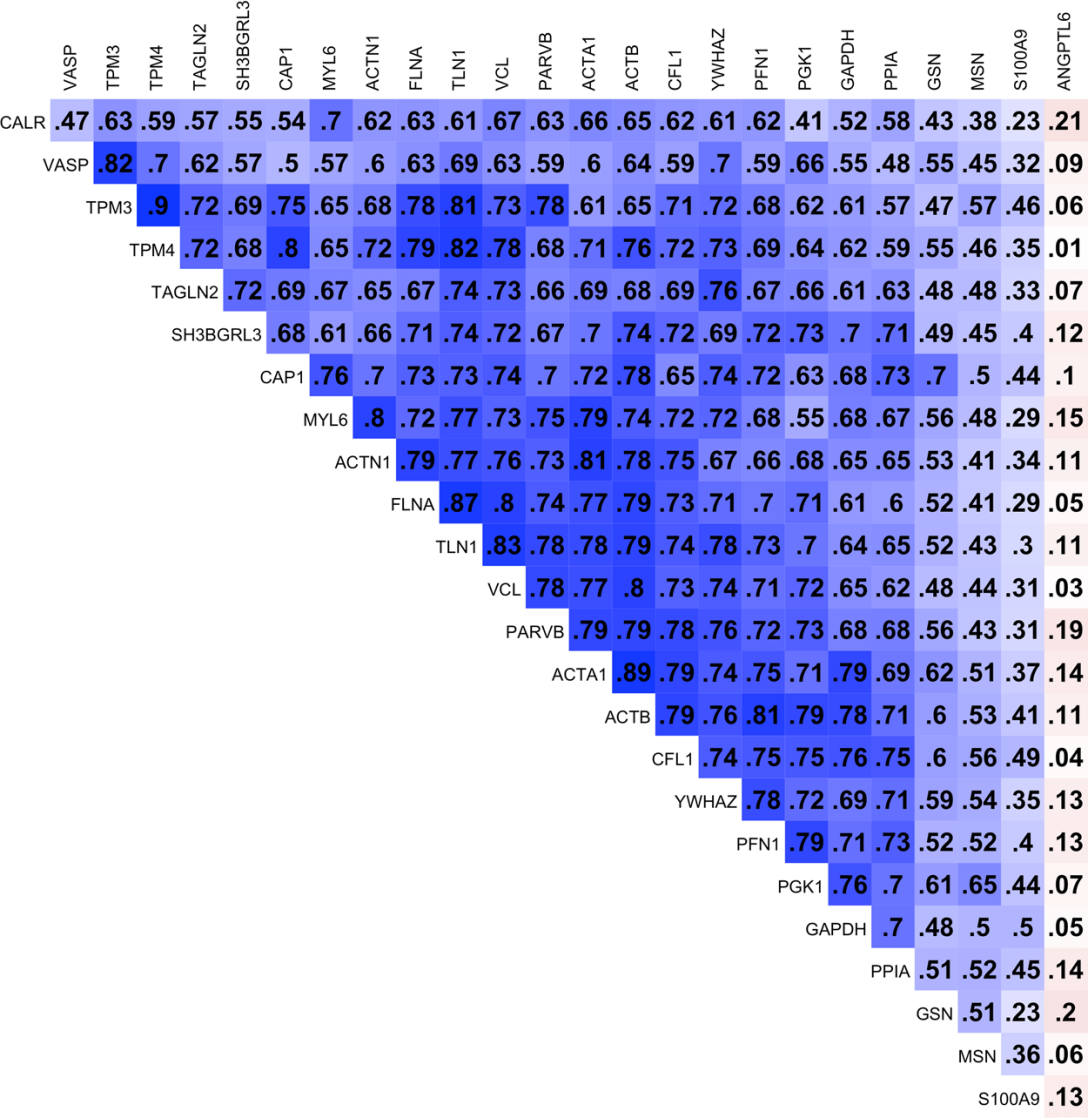
Correlation matrix of plasma proteins differentially abundant between children with small head size and children with normal head size (head circumference-for-age z-scores < −2 or ≥ −2) at birth (q < 0.05). Blue and red color indicate positive and negative correlations, respectively, and strength of association is related to color intensity. Abbreviations: ACTA1, actin, alpha skeletal muscle; ACTB, beta actin; ACTN1, alpha-actinin-1; ANGPTL6, angiopoietin-like 6; CALR, calreticulin; CAP1, adenylyl cyclase-associated protein 1; CFL1, cofilin-1; FLNA, filamin-A; GAPDH, glyceraldehyde-3-phosphate dehydrogenase; GSN, gelsolin; HCZ, head circumference-for-age z-score; MSN, moesin; MYL6, myosin light polypeptide 6; PARVB, beta-parvin; PFN1, profilin 1; PGK1, phosphoglycerate kinase 1; PPIA, peptidylprolyl isomerase A; S100A9, protein S100-A9; SH3BGRL3, SH3 domain-binding glutamic acid-rich-like protein 3; TAGLN2, transgelin-2; TLN1, talin 1; TPM3, tropomyosin alpha-3 chain; TPM4, tropomyosin alpha-4 chain; VASP, vasodilator-stimulated phosphoprotein; VCL, vinculin; YWHAZ, 14-3-3 protein zeta/delta.

The less abundant proteins comprised actin proteins (α- and β-actin); actin-binding proteins that form the actin filament complex (α-actinin, vinculin, talin, parvin, and filamin) and regulate actin cytoskeleton remodeling (cofilin, profilin, and gelsolin); proteins involved in muscle contraction (tropomyosin 3 and 4, transgelin 2, and myosin light polypeptide 6); a chaperone protein (14-3-3 zeta/delta); and glycolytic enzymes [glyceraldehyde-3-phosphate dehydrogenase (GAPDH) and phosphoglycerate kinase 1 (PGK1)]. Except for ANGPTL6, which was a single more abundant protein in children born with small vs. normal head circumference, a correlation matrix among all proteins that were less abundant reveals proteins are highly positively correlated with each other (median r = 0.68; IQR: 0.57 to 0.73) (Fig. 2).

## Discussion

In this typical, rural setting of Nepal, poor prenatal growth manifests as stunting, underweight or wasting at birth, as well as deficits in other less measured dimensions such as a small head circumference. Using an untargeted proteomics approach, we sought to detect plasma proteomic signatures of reduced fetal growth in a generally malnourished population cohort of school-aged children. Our results revealed an absence of quantifiable proteins associated with indicators of newborn weight and length, but a significant differential relative abundance for twenty-five proteins in children born with a small versus normal sized head circumference. These results offer evidence that reduced fetal cranial growth may reflect lastingly altered protein regulation in early life, evident in the plasma proteome at six to eight years of age.

Angiopoietin-like 6 (ANGPTL6) was the only protein whose abundance was elevated in children with a small head circumference at birth. It is a member of an angiopoietin-like family, which is involved in angiogenesis and metabolic homeostasis^24^. Animal studies have shown increased serum ANGPTL6 concentration to be associated with increased energy expenditure and an improved lipid profile and insulin sensitivity^25,26^. However, human studies have reported paradoxical results, with serum ANGPTL6 being elevated in women with pregnancy-induced hypertension^27,28^, diabetic patients^29^ and individuals with metabolic syndrome^30^, suggesting compensatory up-regulation mechanisms. Although results from these few studies are mixed, an elevated plasma ANGPTL6 abundance in children with compromised fetal cranial growth may indicate an early trajectory of disturbed endothelial and metabolic functions, which should be further tested in cohort studies.

Most proteins that were less abundant in children with a small head size at birth were actin or actin-binding proteins. Actin is the most abundant intracellular protein, forming actin filament complex with crosslinkers (α-actinin, vinculin, talin, parvin, and filamin) and assembly/disassembly promotors (cofilin, profilin, and gelsolin)^31^. A chaperone protein, 14-3-3 zeta/delta (YWHAZ)^32^, glycolytic enzymes (GAPDH and PGK1)^33,34^, and peptidylprolyl isomerase A (PPIA)^35^ also showed high positive correlations with other actin-related proteins (Fig. 2), corroborating their known roles in the regulation of cytoskeleton structure. There is little human data that support a hypothesis that changes in intracellular structural composition are associated with inadequate intrauterine growth. Some studies have found typical plasma proteins involved in inflammatory or immune response, nutrient transport, and blood coagulation to be differentially abundant in umbilical cord or venous blood samples between IUGR and non-IUGR newborns^12,14^. The difference between these findings and those reported here may be the result of the depletion process of high abundance proteins carried out in the present study, which allowed us to detect less abundant intracellular proteins in the plasma^36^. It is also possible that the differences in immunologic or metabolic responses between IUGR and non-IUGR neonates may not remain significant in childhood. Experimental animal studies have shown changes in expression of cytoskeleton related proteins in kidney, brain, and small intestine in newborn IUGR offspring^8,9,11^. Swaili *et al*. have reported global changes in gatekeeper genes and proteins, including cytoskeletal proteins in mice embryos, suggesting that cytoskeletal remodeling and cell cycle regulation are the causal mechanisms of nutritional programming^37^. On the other hand, earlier animal studies have suggested that nutritional deprivation in fetal life can disturb cell multiplication and that deficits in tissue or organ cell number are not fully recoverable^38^. These observations lead us to postulate that reduced abundance of the actin filament complex in plasma may reflect reduced cellularity or impaired cell differentiation of vulnerable tissues, or epigenetic regulation of cell structure and cycle related gene expression^39^. The fundamental roles of the cytoskeleton in early developmental processes and cell physiology and fate may provide a platform to mediate prenatal effects on postnatal life.

Because identified proteins were specific in their association with head size but not with other body size parameters at birth, one might consider that affected proteins could reflect neurological impairment in the brain. Head circumference at birth is well correlated with brain growth in newborns^40^ and has shown positive associations with cognitive abilities of children in some studies^41,42^. For example, actin cytoskeleton plays a critical role in developmental processes of the brain including neurite outgrowth, proliferation, and migration^43^. Disruption of actin cytoskeleton in the brain is associated with microcephaly and abnormal cortical development^44^. In the larger cohort of the same children in this study, head circumference at birth was positively associated with test scores of general intelligence, executive function, and motor function^45^. However, in a separate analysis, we found no association between the proteins observed to be related to a small head circumference and performance on these same cognition tests^23^. As ANGPTL6 is mainly a liver-derived protein and tropomyosin 3 and 4 are abundant in muscle^46^, the brain might not be the only organ that was affected by prenatal exposures. Because actin cytoskeleton, for example, is ubiquitous, it is possible that the identified proteins may reflect systemic changes in peripheral tissue proteomes in response to probably the most severe nutritional deficits during early life in this study population. Tissue origins and physiological and clinical significance of the identified proteins need further investigation.

To the best of our knowledge, this is the first comparative plasma proteomics study in human subjects that has examined enduring effects of restricted fetal growth on a plasma proteome in mid-childhood. Rigorous methods of pregnancy assessment and repeated birth anthropometry within 72 hours of birth under rural field settings^15^ increase confidence in the reliability of neonatal measurements. In the laboratory, our strategies of random sampling and assignment of plasma samples to mass spectrometry channels and experiments minimized chances of contamination or experimental artifacts^47^. An untargeted and high-throughput proteomics approach offered by mass spectrometry allowed detection of subtle changes in multiple individual proteins that are functionally coherent, strengthening the validity of our findings. Among limitations, although we adjusted for extensive variables of maternal pregnancy, child characteristics and household socioeconomic status in our models, the possibility of residual confounding cannot be ruled out. Because proteins were quantified on a relative scale, absolute changes in plasma abundance have not yet been possible to measure. Lastly, proteomics data observed at a single time point is insufficient to definitively discern whether observed differences are transient or persistent. Further cohort assessments at older ages will be required to ascertain whether suppressed or overexpressed protein differences are sustained into adulthood and whether these patterns are associated with functional or health outcomes.

In this systematic exploration of the plasma proteome, we identified a novel cluster of biomarkers associated with a constrained head size in a South Asian population of school-aged children. As affected proteins may be expected to vary by population exposure, phenotype and proteomics methods employed, these findings should be considered preliminary and in need of verification in other birth cohorts. Further studies are warranted to examine clinical and public health implications of plasma proteomic patterns associated with growth, nutrition and other exposures early in life.

## Methods

### Study population and design

In a community-based trial conducted from 1999 to 2001 in Sarlahi District, located in rural Southeastern Nepal, nearly 5,000 pregnant women were randomized to receive from early pregnancy through 12 weeks postpartum daily antenatal micronutrient supplements containing vitamin A alone as the control or folic acid, iron-folic acid, iron-folic acid-zinc, or multiple micronutrients^15^. In this trial, iron-folic acid and multiple micronutrient supplements improved multiple dimensions of birth size and reduced the risk of low birthweight compared to the control. Children born to mothers who had participated in the trial were then followed-up in 2006–2008, when they were 6–8 years of age^16,48^. Children in the present plasma proteomics study were a subset of the larger child cohort. Full details of this sub-study sample, study design, sampling strategies have been published elsewhere^17^. Briefly, among 3,524 children assessed at the time of the follow-up, 2,130 children met our sampling frame criteria (i.e., availability of sufficient plasma volumes, complete epidemiological data collected during both the maternal trial and child follow-up assessment, and birth size measures obtained within 72 hours after birth). These children were stratified into one of five maternal micronutrient supplementation groups, from which 1000 were randomly selected, 200 per maternal trial supplement group, for extensive biochemical nutritional analyses^49^. From each stratum, we randomly selected a 50% sample, or 100 children per maternal trial group, for plasma proteomics analysis. The original maternal micronutrient supplementation trial was registered at ClinicalTrials.gov as NCT00115271. Due to high illiteracy in the study population, oral informed consent was obtained from parents of eligible children by trained field staff during the child follow-up. Ethical approval for both maternal and child follow-up studies was obtained from the institutional review board at Johns Hopkins University, Baltimore, MD, USA and the Nepal Health Research Council in Kathmandu, Nepal. All methods were carried out in accordance with the approved guidelines and regulations.

### Birth assessment

Because most women delivered at home, birth anthropometry was conducted by trained anthropometrists during a home visit^15^. All birth anthropometry data in this study was collected within 72 hours of birth. Birth weight was measured to the nearest 2 g using a digital scale. Recumbent length was determined in triplicate to the nearest 0.1 cm on a length board. Head circumference was measured in triplicate to the nearest 0.1 cm with an insertion tape. We used the median of the three values of length and head circumference and calculated z-scores for weight-for-age (WAZ), length-for-age (LAZ), weight-for-length (WLZ), and head circumference-for-age (HCZ) based on the World Health Organization (WHO) child growth standards^50^. Implausible z-scores (LAZ < −6, n = 1; WLZ < −5, n = 1; HCZ < −5, n = 3) and unavailable z-scores for WLZ (recumbent length < 45 cm, n = 41) were treated as missing^51^. Children with z-scores less than −2 were considered to be born with small sizes, compared to the reference population of the WHO growth standards. We classified newborn size as normal or small, with the latter being <−2 in WAZ, LAZ, WLZ, and HCZ. Maternal data including age and parity during pregnancy was collected at the 1^st^ trimester of pregnancy. Gestational age was estimated based on the first day of last menstrual period.

### Child follow-up assessment & blood sample collection

Child characteristics (e.g., literacy, attained years of schooling) and household socio-economic status (e.g., asset ownership, ethnicity, caste and head of household education) were collected during the follow-up study^16^. The same team of trained anthropometrists as during the maternal trial visited children in their homes to measure child weight, height, and left mid-upper arm circumference following standard procedures. Height-for-age, weight-for-age and body mass index [BMI, weight (kg) / height^2^ (m)]-for-age z-scores were calculated based on the WHO growth reference^52^. On the following morning of the anthropometry assessment, field phlebotomists visited the homes and collected overnight-fasted venous blood samples from children^48^. Biospecimens were brought to the field laboratory for plasma extraction, stored and shipped in dry liquid nitrogen tanks to the Center for Human Nutrition, Johns Hopkins Bloomberg School of Public Health, Baltimore, USA where they were stored −80 °C freezers until thawed for analyses.

### Plasma proteomics

Plasma proteomics analysis procedures have been previously reported^17^. Briefly, six high-abundance proteins (albumin, haptoglobin, immunoglobulin A and G, transferrin, and anti-trypsin), comprising 85% of total plasma proteins, were removed from each of 500 40 μl plasma samples for enhancing detection sensitivity of low abundance proteins using a Human 6 multiple affinity removal system column (Agilent Technologies, California, USA)^36^. Depleted plasma samples (each containing ~100 μg of protein) were treated with trypsin overnight for protein digestion. Peptide samples from 7 individual samples with one pooled sample (internal standard) were randomly labeled with 8-plex isobaric Tag for Relative and Absolute quantitation (iTRAQ) reagents (AB Sciex), which contain different reporter ions. The eight samples were combined and separated by strong cation exchange chromatography into 24 fractions. Each fraction of labeled peptide samples was analyzed by mass spectrometry using an Eksigent 2D nano LC interfaced with a LTQ Orbitrap Velos mass spectrometer (Thermo Scientific). Peptides were identified by searching precursor and fragment mass data against the Refseq 40 protein database using MASCOT (Matrix Science) through Proteome Discoverer software (v1.3, Thermo Scientific). Peptide identification was performed with a confidence threshold of <5% false discovery rate. A total of 72 iTRAQ 8-plex mass spectrometry experiments were run for all plasma samples of children (n = 500).

### Statistical analyses

Statistical methods of protein relative abundance quantification from the iTRAQ reporter ions were previously reported^47^. Briefly, the relative abundance of proteins in each channel of each iTRAQ experiment was estimated by computing the median of all the median-polished log2-transformed iTRAQ reporter ion intensities across all spectra belonging to each protein. Varying numbers of missing values were observed across proteins and unobserved values were considered to be missing at random^47^. We estimated mean differences in relative abundance of proteins between two groups of children classified by small vs. normal size at birth. We employed linear mixed-effects models with each protein as a dependent variable, each dichotomized birth size group as a fixed effect, and iTRAQ experiment as a random effect to take into account any random effects that can be derived from extreme values. P-values were calculated by using a two-sided test of a null hypothesis that there is no difference in protein relative abundance between two groups. We estimated q-values to control a false discovery rate (FDR) and considered proteins passing a FDR threshold <5% (q < 0.05) as being significantly differentially abundant^53^. We considered child age, sex, height, body mass index, and schooling as covariates. We identified household ethnicity, caste, and wealth index, and maternal age and parity during pregnancy, and gestational age as potential confounding factors and adjusted for them. The household wealth index variable was created by calculating the 1st principal component of the polychoric correlation of selected items of household assets (construction materials of ground floor, first floor, and roof of house, bicycle, radio, television, electricity, cattle, goat, and land ownership). Maternal micronutrient supplementation during pregnancy was not included in the adjusted model due to its having no effects on child plasma proteome (Lee SE *et al*., unpublished data, 2017). We report adjusted differences in relative abundance of proteins between small vs. normal size at birth and unadjusted estimates are listed in Supplementary Table S2. We plotted volcano plots to display all analyzed plasma proteins with corresponding adjusted percentage differences in relative abundance of proteins (%) in the x-axis and statistical significance (−log_10_p-value) in the y-axis.

We built a correlation matrix of proteins associated with small size at birth to examine biological relationships among the associated proteins. Because proteins were quantified as relative abundance within each iTRAQ experiment, we calculated protein-protein *Pearson* correlation coefficients using complete pairwise data in each mass spectrometry experiment, and used the averaged coefficients across all experiments. The order of proteins was determined by optimal leaf ordering that organizes more correlated elements adjacent.

### Functional analysis

To identify statistically over- or under-represented functional clusters in the list of proteins differentially abundant between small vs. normal size at birth, we conducted an over-representation test using the PANTHER (Protein Annotation Through Evolutionary Relationship) classification system (version 11.1. Released 2016-10-24)^54^. For protein annotation, we used defaulted PANTHER Gene Ontology (GO)-Slim datasets, which are hierarchically comprised of GO terms in three aspects: molecular function, cellular localization, and biological process^55^. Identified proteins associated with small birth size were used as an input analyzed list and all proteins quantified by mass spectrometry detected in >10% of study children were used as an input reference list. Numbers of classified proteins in analyzed and reference lists are compared in each functional cluster. P-values were calculated by Binomial statistics under a null hypothesis that identified proteins associated with small size at birth are sampled from the same general population as proteins from the reference set^56^. Annotation categories with Bonferroni-corrected p-value < 0.05 were considered statistically significant.

### Data availability

The datasets of birth anthropometry and relative abundance of proteins included in this published article are available in Supplementary Table S3. All analyses were performed by using the R Environment for Statistical Computing (version 3.1.2; R Development Core Team).

## Electronic supplementary material

Online supplementary Table 1-2

Online supplementary Table 3 (Dataset 1)
