# Supplementary material for: Novel Plasma Proteins in Nepalese School-aged Children are Associated with a Small Head Size at Birth
Source: Sci Rep. 2018 Apr 23;8:6390. doi: 10.1038/s41598-018-24640-4 (PMC5913316; doi:10.1038/s41598-018-24640-4)
Supplement: Supplementary file 1 — Online supplementary Table 1-2 [file 41598_2018_24640_MOESM1_ESM.pdf]

Novel Plasma Proteins in Nepalese School-aged Children Are Associated with a Small Head Size at Birth

Sun Eun Lee[1], Keith P. West, Jr[1], Robert N. Cole[2], Kerry J. Schulze[1], Lee S-F Wu[1], James D. Yager[3], John Groopman[3], Parul Christian[1]

[1]Center for Human Nutrition, Dept. of International Health, Johns Hopkins Bloomberg School of Public Health, Baltimore, MD 21205, U.S.A.

[2]Mass Spectrometry and Proteomics Facility, Department of Biological Chemistry, Johns Hopkins School of Medicine, Baltimore, MD 21205, U.S.A.

[3]Department of Environmental Health and Engineering, Johns Hopkins Bloomberg School of Public Health, Baltimore, MD 21205, U.S.A.

Table S1. Characteristics of children at the time of blood draw for proteomics analysis (n=500).

	Value
**Child characteristics**	
Girl, %	50.2
Age, years	7.5 (0.4)
Ethnicity[a], Madheshi, %	68.2
Ever attended school, %	66.8
**Child anthropometric measurements[b]**	
Weight, kg	18.2 (2.2)
Height, cm	114.1 (5.8)
MUAC, cm	15.4 (1.1)
Body mass index (BMI), kg/m^2	14.0 (1.0)
**Child undernutrition[b,c], %**	
Stunted (height-for-age z-score < -2)	39.1
Underweight (weight-for-age z-score < -2)	48.5
Low BMI (BMI-for-age z-score < -2)	16.4
**Caste, %**	
Brahmin or Chhetri	14.0
Vaiysha	65.2
Shudra, Muslim or Buddhist	20.8
**Household asset, %**	
Land ownership	77.0
Bicycle	61.2
Cattle	69.6
Goat	65.6
Electricity	51.0
Television	30.4

Data are expressed as mean (standard deviation) or %. [a]Ethnicity was classified as either Madheshi (originating from the plains) or Pahadi (originating from the hills). [b]One outlier was excluded. [c]Anthropometry z-scores were calculated based on the WHO reference for children 5-19 years of age.

Table S2. Plasma proteins differentially abundant between children born with small head size and children born with normal head size (Head circumference-for-age z-scores < -2 or ≥ -2), q<0.05.

Protein name[a]	Gene symbol	n for HCZ ≥ -2[b]	n for HCZ < -2[b]	% Difference (95% CI)[c]	p-value[d]	q-value[e]	Accession[f]
14-3-3 protein zeta/delta	YWHAZ	294	78	-15.0 (-20.8, -8.9)	4.03×10^{-6}	0.0034	21735625
Transgelin-2	TAGLN2	327	87	-15.1 (-21.2, -8.5)	1.69×10^{-5}	0.0063	4507357
Actinin, alpha 1	ACTN1	370	92	-12.9 (-18.3, -7.1)	2.27×10^{-5}	0.0063	194097352
Talin 1	TLN1	393	97	-11.5 (-16.4, -6.3)	2.94×10^{-5}	0.0063	223029410
SH3 domain-binding glutamic acid-rich-like protein 3	SH3BGRL3	373	89	-15.3 (-21.9, -8.2)	5.35×10^{-5}	0.0082	13775198
Vasodilator-stimulated phosphoprotein	VASP	264	63	-15.5 (-22.2, -8.3)	5.77×10^{-5}	0.0082	4507869
Angiopoietin-like 6	ANGPTL6	148	47	16.5 (7.6, 26.0)	0.0001	0.0168	29893555
Tropomyosin alpha-4 chain	TPM4	303	82	-14.9 (-21.7, -7.4)	0.0002	0.0172	4507651
Vinculin	VCL	396	101	-8.5 (-12.8, -4.0)	0.0003	0.0256	4507877
Tropomyosin alpha-3 chain	TPM3	114	39	-20 (-29.2, -9.6)	0.0003	0.0258	114155146
Gelsolin	GSN	277	76	-8.4 (-12.8, -3.9)	0.0004	0.0293	38044288
Filamin-A	FLNA	396	101	-8.6 (-13.1, -3.9)	0.0004	0.0293	116063573
Calreticulin	CALR	265	72	-7.0 (-10.8, -3.1)	0.0005	0.0330	4757900
Moesin	MSN	339	88	-6.2 (-9.5, -2.7)	0.0005	0.0330	4505257
Adenylyl cyclase-associated protein 1	CAP1	139	40	-17.3 (-25.9, -7.7)	0.0007	0.0353	5453595
Calponin-2	CNN2	43	12	-24.8 (-36.5, -11)	0.0007	0.0353	41327730
Beta actin	ACTB	396	101	-8.7 (-13.4, -3.7)	0.0007	0.0353	4501885
Glyceraldehyde-3-phosphate dehydrogenase	GAPDH	390	100	-10.2 (-15.6, -4.4)	0.0007	0.0353	7669492
Phosphoglycerate kinase 1	PGK1	103	30	-17.9 (-26.9, -7.7)	0.0008	0.0360	4505763
Parvin, beta	PARVB	235	63	-18.3 (-27.4, -7.9)	0.0008	0.0360	20127528
Cofilin-1	CFL1	266	72	-11.5 (-17.6, -4.8)	0.0009	0.0369	5031635
Myosin light polypeptide 6	MYL6	243	57	-16.3 (-24.8, -6.9)	0.0010	0.0391	17986258
Profilin 1	PFN1	368	96	-10.5 (-16.2, -4.3)	0.0011	0.0391	4826898
Actin, alpha skeletal muscle	ACTA1	292	75	-11.7 (-18.1, -4.7)	0.0013	0.0481	4501881

Abbreviations: HCZ, head circumference z-scores. [a]Proteins are listed in increasing order of q. [b]Data were missing for HCZ (n=3). [c]Percent difference (95% confidence interval) in relative abundance of protein between children born with small compared to normal head circumference. [d]*P* value was calculated by testing a null hypothesis of no difference in protein relative abundance between two groups. [e]Multiple hypothesis testing was corrected using false discovery rate. [f]GenInfo sequence number as assigned to all nucleotide and protein sequences by the National Center for Biotechnology Information at the National Library of Medicine, NIH.
